# Effect of vitamin D on renin concentration in chronic heart failure patients: a randomized placebo-controlled trial

**DOI:** 10.21542/gcsp.2025.7

**Published:** 2025-02-28

**Authors:** Fathia Mghaieth, Bessem Hammami, Syrine Ben Jeddou, Selim Boudiche, Manel Ben Halima, Jihen Bensassi, Amine Soula, Sameh Hadj Taieb, Moncef Feki, Mohamed S. Mourali

**Affiliations:** 1 University of Tunis El Manar, Faculty of Medicine of Tunis, Tunis, Tunisia; 2 Rabta Hospital, Service of Cardiology, 1007 Jebbari, Tunis, Tunisia; 3 Rabta Hospital, Laboratory of Biochemistry & LR99ES11, 1007 Jebbari, Tunis, Tunisia

## Abstract

**Background:** Hypovitaminosis D and hyperreninemia are associated with poor prognosis in patients with chronic heart failure (CHF). We aimed to evaluate the effect of vitamin D (VitD) supplementation on the renin-angiotensin-aldosterone system (RAAS) in CHF patients with reduced left ventricular ejection fraction (HFrEF).

**Methods:** A double-blind placebo-controlled randomized clinical trial was conducted in HFrEF patients. Patients were randomized to receive two doses of 200,000 IU VitD3 (VitD group) or saline solution (placebo group) at a two-week interval. Plasma 25-hydroxyvitamin D and renin concentrations were assessed at baseline and one month after the first dose. The primary outcome was plasma renin change. The study was registered at the Pan African Clinical Trials Registry as PACTR201908774181973.

**Results:** Forty patients in each group completed the trial. At baseline, hypovitaminosis D and hyperreninemia were found in 85% and 77.5% of patients, respectively, with no differences between VitD and placebo groups. VitD supplementation resulted in an increase in 25-hydroxyvitamin D in the VitD group, only. In parallel, plasma renin decreased in the VitD group [renin change: median (25th percentile; 75th percentile), −34.9 (−110; −7.1)], but did not change in the placebo group [1.35 (−1.2; 8.8)]. No significant changes were found for clinical, echocardiographic, and other biochemical parameters in both groups.

**Conclusions:** A mega-dose of vitamin D resulted in middle-term RAAS suppression but didn’t affect cardiac function and clinical features in HFrEF patients. Further research with longer follow-ups is needed to evaluate whether persistent adequate VitD status results in long-term RAAS suppression and cardiac function improvement.

## Introduction

Chronic heart failure (CHF) is a major public health problem. Its prevalence in adults is estimated at 1–2% and reaches more than 10% over 70 years^1^. This prevalence is increasing, due to the aging of the population and the better management of ischemic, hypertensive, and valvular heart diseases^2^. The renin-angiotensin-aldosterone system (RAAS) plays a central role in CHF and its inappropriate activation appears to be responsible for the progression of this disease^3^. For this reason, the modulation of this system has become a major target for CHF treatment. Some studies have shown that despite their proven efficacy in terms of morbidity and mortality, the usual drugs for CHF acting on the RAAS (such as ACE inhibitors, anti-aldosterone, and neprilysin-ACE inhibitor combination) lead to an increase in the activity as well as the concentration of plasma renin^4^. Such an increase has been associated with poor prognosis and high mortality in patients with CHF^5^.

Vitamin D (VitD) is a fat-soluble vitamin and considered a real hormone. Hypovitaminosis D has been associated with an increased risk of hypertension, ischemic heart disease, and stroke, and has been implicated in the physiopathology of CHF^6^. Several randomized controlled trials (RCTs) have investigated the effect of VitD supplementation using various regimens of supplements (low/high, daily/weekly/monthly doses) in patients with CHF.

A meta-analysis by Zhao et al. ^7^ showed that VitD supplementation inhibits ventricular remodeling and improves cardiac function in patients with HF. This effect is thought to be mediated by the modulation of the RAAS. The hypothesis has been put forward based on experimental studies showing that VitD inhibits the transcription of renin, thus reducing its concentration and plasma activity in animals^8–11^. Data on the direct effect of VitD on RAAS, specifically on renin levels in CHF, are rare and inconsistent^12–14^. In CHF, a maximal activation of the RAAS might make it insensitive to the effect of VitD. The current study evaluated the impact of VitD supplementation on renin levels and clinical and biochemical variables in CHF patients with reduced left ventricular ejection fraction (HFrEF).

## Material and Methods

### Study population

A randomized double-blind clinical trial involved patients from the Cardiology department of Rabta Hospital (Tunis, Tunisia) between January 2020 and June 2022. Eligibility criteria were patients with CHF regardless of the date of diagnosis and the etiology, age over 18, left ventricular ejection fraction (LVEF) <40%, clinically stable or stabilized heart failure [dyspnea stage I or II of New York Heart Association (NYHA), systolic blood pressure ≥ 90 mmHg, and absence of signs of peripheral hypoperfusion. Patients with acute cardiac decompensation or cardiogenic shock, chronic kidney disease (creatinine clearance <15 mL/min), blood calcium ≥ 2.6 mmol/L, known calcium lithiasis, baseline VitD level >50 ng/mL, or receiving vitamin supplements within 6 months were not included in the study. The National Ethics Committee approved the study protocol and participants gave written informed consent. The study was registered at the Pan African Clinical Trials Registry as PACTR201908774181973.

### Study protocol

Patients with HFrEF were recruited from the Cardiology department at Rabta Hospital (Tunis, Tunisia). On the first visit, a clinical exam, a 6-minute walk distance (6-MWD) test, and echocardiography were assessed before the patient’s inclusion. An independent care provider (the head nurse) used an automated computer system to randomly assign eligible patients in a 1:1 ratio into a VitD or placebo (PLB) group.

Patients in the VitD group received a first oral dose of VitD3 (vial of one mL containing 200,000 IU cholecalciferol; Bouchara Recordati Laboratories, Milano, Italy). Patients in the PLB group received a single oral dose of 1 saline solution (vial of one mL of NaCl for injectable preparations (Medis, Nabeul, Tunisia). The head nurse administered the supplement at the discretion of patients, care providers, and investigators. A second medical visit including a physical examination and an electrocardiogram were performed 15 days after the first dose to look for signs of VitD toxicity (i.e., headache, nausea, vomiting, asthenia, confusion or seizure, bradycardia, diminished deep tendon reflexes, PR>200 ms, QT interval <300 ms, QRS>120 ms, and electrical signs of atrioventricular heart block) and an allergic reaction to the treatment. A 24-hour urine sample was collected. Patients with no adverse effects and urinary calcium below 250 mg/24 h received a second treatment dose identical to the supplement previously delivered.

Patients undertook a clinical exam, a 6-MWD test, and an echocardiography. VitD toxicity signs were reassessed. Blood and urine samples were collected at baseline, and 15 and 30 days after the first dosing charge. Allocation remained blind to investigators, care providers, and patients until the end of the study. The usual medication for heart failure was maintained unchanged throughout the trial.

The primary endpoint was the change in plasma renin concentration from baseline to one month after the first dose. Secondary endpoints were changes in clinical, echocardiographic, and biochemical variables (i.e., blood pressure, heart rate, NYHA class, 6-MWD test, LVEF, and plasma 25-OHD and brain natriuretic peptide (BNP) levels). The duration of the trial was adjusted to the biological primary endpoint.

**Figure 1.**
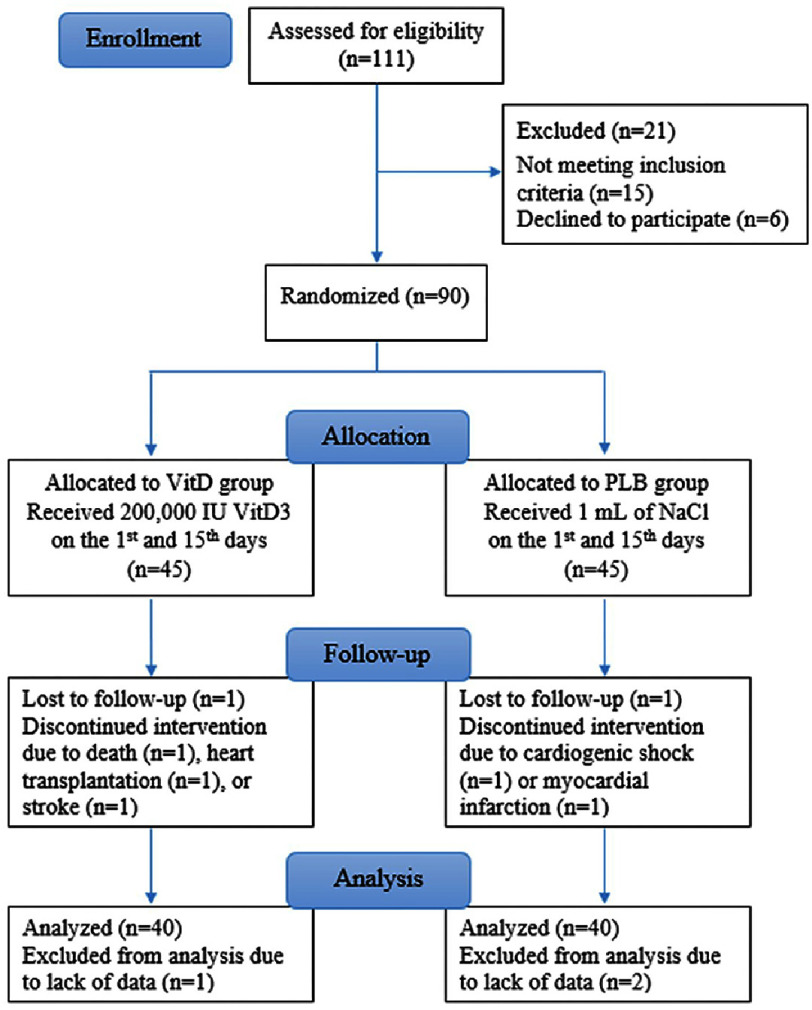
Flowchart of the study participants.

Based on literature data and personal experience, we estimate that oral ingestion of 200,000 IU VD3 at two-week intervals ensures restoring and maintaining accurate vitamin D status at one month even in VitD deficient individuals. Therefore, we deemed this time frame adequate to produce significant renin change if the vitamin affects RAAS. Of ninety CHF patients initially included, 10 patients were excluded due to death, myocardial infarction, cardiogenic shock, heart transplantation, stroke, or lack of data. Finally, 40 patients in each group were retained for the analysis (Figure 1).

### Sample size calculation

A pilot study involving the 20 first patients included in the trial showed that the change in renin concentrations of matched pairs is normally distributed with a mean of 67 pg/mL and a standard deviation of 127 pg/mL. We need to study 40 pairs of subjects to be able to reject the null hypothesis with a probability (power) of 0.9 and a type I error probability of 0.05. To account for potential losses during follow-up, we included 90 patients in the trial.

### Laboratory measurements

Routine parameters including plasma and urine calcium, creatinine, electrolytes, and BNP were analyzed on an Architect Ci8000 analyzer using respective reagent kits (Abbott Laboratories, Abbott Park, IL). Additional plasma samples were stored at −40 ° C for subsequent analyses including plasma 25-hydroxyvitamin D (25-OHD), and renin. Analyses were carried out by radioimmunoassay using commercial reagent kits; Active^®^ Renin IRMA kit for renin and 25-OHD total RIA kit for 25-OHD (Beckman Coulter Diagnostics, Fullerton, CA). All biochemical parameters were analyzed in the laboratory under internal and external quality control, aligning with international standards. VitD insufficiency was defined by plasma 25-OHD <30 ng/mL, and hyperreninemia was defined for renin values >20 pg/mL^15^. The estimated glomerular filtration rate (eGFR) was calculated using the 4-point Modification of Diet in Renal Disease (MDRD) formula.

### Statistical analysis

Statistical analysis was performed using SPSS 18.0 software (SPSS Inc, Chicago, Il). Categorical variables were reported as a percentage of observations. A comparison of percentages was carried out using the Pearson Chi^2^ test or Fisher test, as appropriate. For quantitative variables, the normality of the distribution was tested using the Shapiro–Wilk test. Normally distributed variables were represented as means with standard deviation. Comparisons of continuous variables between groups were conducted using independent samples t-tests or Mann–Whitney tests, as appropriate. Two-way repeated measure ANCOVA models were applied (with time as a within-subjects factor and supplementation as a between-subjects factor) to examine the interaction effect for the group (VitD/PLB groups) by time (pre-/post-supplementation) for the primary and secondary endpoints. Covariates were age, gender, NYHA class, baseline LVEF, and plasma 25-OHD concentrations. If a significant interaction was detected, within-group comparisons between pre- and post-supplementation values were conducted using dependent sample t-tests or Wilcoxon rank tests for non-normally distributed variables (i.e., BNP and renin). A two-tailed *p*-value of less than 5% was considered significant.

## Results

### Baseline characteristics of participants

Baseline characteristics, specifically plasma 25-OHD and renin concentrations did not differ between the VitD and PLB groups. Most patients (90% and 85% in VitD and PLB groups) have hypovitaminosis D (25-OHD<30 ng/mL). Increased renin concentrations were found in 80% and 75% of VitD and PLB groups, respectively (Table 1).

**Table 1:** Baseline characteristics of HFrEF patients in vitamin D and placebo groups.

Characteristic	PLB group (*n* = 40)		VitD group (*n* = 40	*P*-value	
Clinical and echocardiographic characteristics				
Age (years)		60.3 ± 14.1	61.8 ± 10.9		0.61
Male gender (%)		70.0	87.5		0.09
Systolic blood pressure (mm Hg)		124 ± 22.2	124 ± 20.1		0.94
Diastolic blood pressure (mm Hg)		68.3 ± 12.3	71.0 ± 12.5		0.34
Heart rate (beat/min)		76.2 ± 16.0	73.5 ± 12.7		0.39
Ischemic etiology (%)		75.0	85.0		0.40
NYHA class II (%)		92.5	85.0		0.48
6-minute walk distance (min)		320 ± 137	358 ± 166		0,27
LVEF (%)		28.5 ± 8.5	31.6 ± 6.5		0.07
eGFR (mL/min/1.73 m^2^ )		77.2 ± 20.4	79.9 ± 24.9		0.60
Medications				
ACEis/ARBs (%)		82.5	85.0		0.79
Beta Blockers (%)		87.5	87.5		1.00
Aldosterone antagonist (%)		45.0	40.0		0.82
Loop diuretic (%)		57.5	57.5		1.00
Laboratory parameters				
Creatinine (mg/L)		10.4 ± 2.8	10.7 ± 3.3		0.69
Calcium (mg/L)		90.2 ± 6.1	90.2 ± 5.9		0.99
Urinary sodium (mmol/L)		77.2 ± 43.5	74.8 ± 43.4		0.80
Urinary potassium (mmol/L)		54.6 ± 28.3	58.8 ± 35.5		0.56
Brain natriuretic peptide (pg/mL)		100 (63-584)	134 (45-253)		0.47
25-hydroxyvitamin D (ng/mL)		13.4 ± 7.2	13.9 ± 9.3		0.78
Renin (pg/mL)		49 (17-192)	63 (23-205)		0.95

**Notes.**

ACEis angiotensin-converting enzyme inhibitors ARBs angiotensin II receptor blockers eGFR estimated glomerular filtration rate HFrEF heart failure with reduced ejection fraction LVEF left ventricular ejection fraction NYHA New York Heart Association PLB placebo VitD vitamin D

### Intervention-induced changes

**Figure 2.**
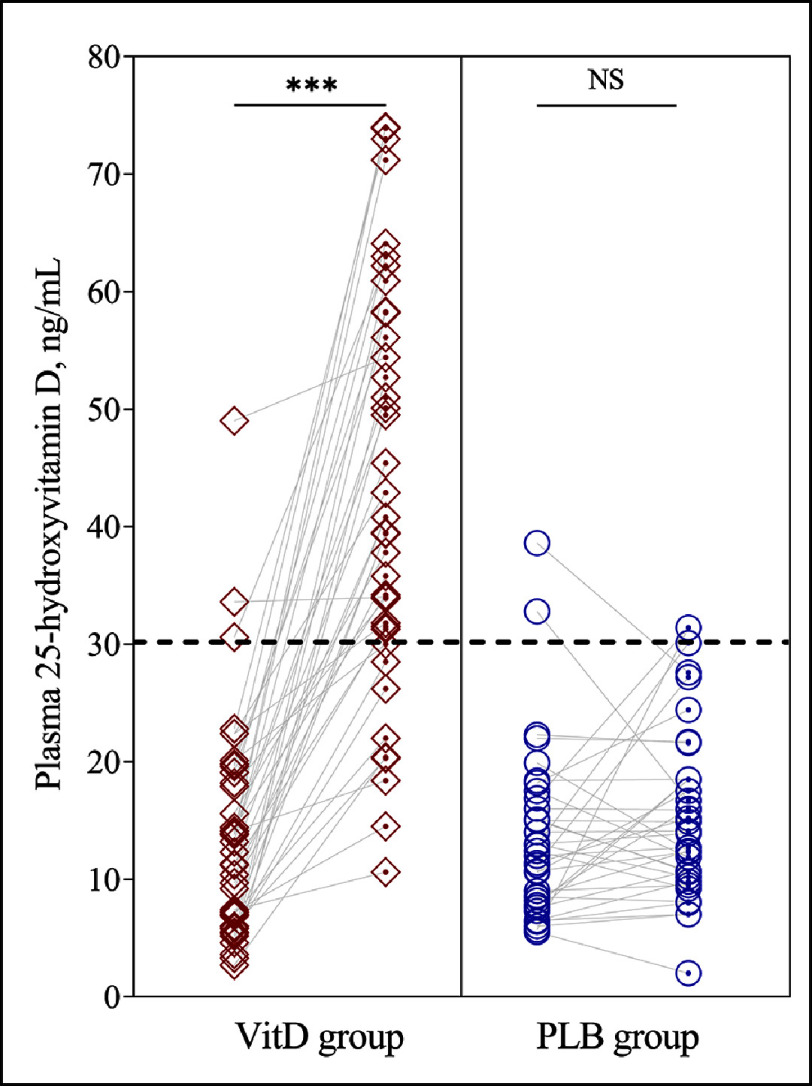
Changes in plasma 25-hydroxyvitamin D from baseline to the end of the follow-up in vitamin D (VitD) and placebo (PLB) groups. The black discontinued line refers to the normal cut-off value.

**Figure 3.**
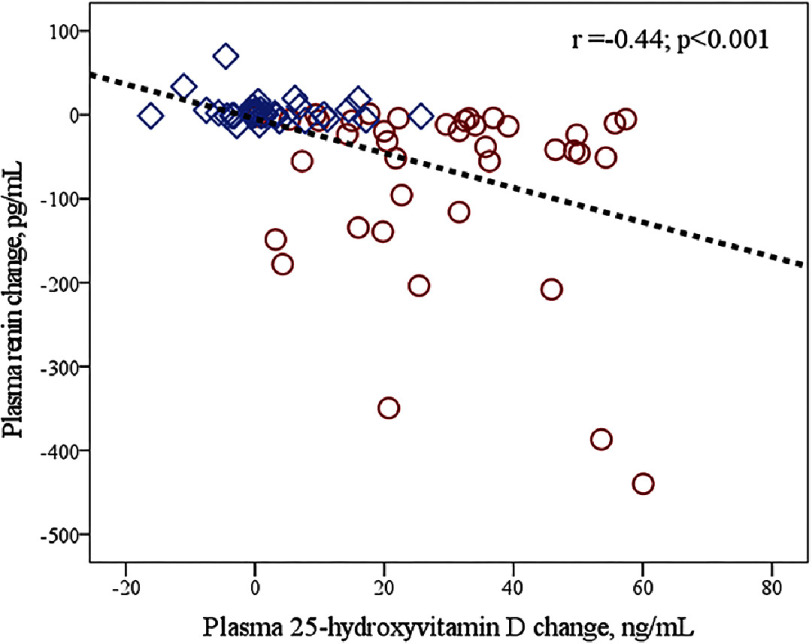
Scatter plots showing correlations between plasma renin change and plasma 25-hydroxyvitamin D change in vitamin D (circles) and placebo (diamonds) groups.

**Figure 4.**
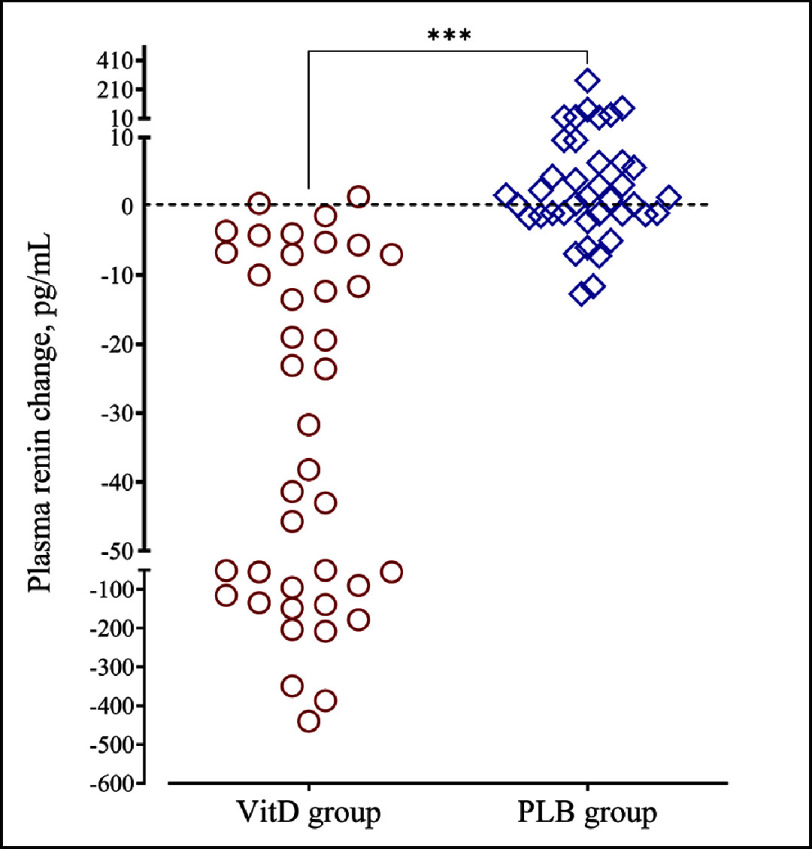
Change in plasma renin concentrations from baseline to the end of the follow-up in vitamin D (VitD) and placebo (PLB) groups.

Repeated measure analyses showed significant “time*group” interactions for plasma 25-OHD (*F* = 61.6; *P* < 0.001; power, 1.00) and for plasma renin (*F* = 16.6; *P* < 0.001; power, 0.98). VitD supplementation significantly increased 25-OHD concentrations while no significant change was found in the PLB group (Table 2, Figure 2). At the end of the trial, VitD sufficiency was achieved in 77.5% and 17.5% of patients in the VitD and PLB groups, respectively. In parallel to these changes, plasma renin concentrations decreased in the VitD group but did not change in the PLB group (Table 2, Figure 3). Plasma renin changes were meaningly more important in the VitD group (ranging from −470 to +1.42 pg/mL) than in the PLB group (ranging from −13.1 to +270 pg/mL). Moreover, renin changes were inversely related to 25-OHD changes (Figure 4).

**Table 2:** Pre- and post-supplementation values and interaction time*group for clinical, and biochemical measures in vitamin D and placebo groups.

	Placebo group (*n* = 40)			Vitamin D group (*n* = 40)			Interaction (time*group)^a^	
	Pre	Post		Pre	Post		F coefficient	Observed power
Renin (pg/mL)	49 (17-192)	54.7 (21-259)		63 (23-205)	22.5 (9-64) ^***^		16.6 ^###^	0.98
25-OHD (ng/mL)^b^	13.4 ± 7.2	15.7 ± 7.35		13.9 ± 9.3	43.1 ± 17.4 ^***^		61.6 ^###^	1.00
BNP (pg/mL)	100 (63-584)	103 (31-268)		134 (45-253)	168 (59-350)		1.042	0.17
SBP (mm Hg)	124 ± 22.2	121 ± 19.1		124 ± 20.1	123 ± 18.1		0.045	0.06
DBP (mm Hg)	68.3 ± 12.3	66.1 ± 11.0		71.0 ± 12.5	67.0 ± 11.3		0.027	0.36
Heart rate (beat/min)	76.2 ± 16.0	74.5 ± 11.7		73.5 ± 12.7	73.1 ± 9.65		0.693	0.13
6-MWD (min)	320 ± 137	314 ± 138		358 ± 166	373 ± 171		2.992	0.40
LVEF (%)^c^	28.5 ± 8.5	30.4 ± 9.50		31.6 ± 6.5	34.9 ± 9.74		0.368	0.09

**Notes.**

Data are expressed as mean ± SD or median (25^th^ –75^th^ percentile).

*** *P* < 0.001 (Within-group comparisons were achieved using paired-samples t-test or Wilcoxon rank test with the pre-supplementation value as a reference).

a Interaction was tested using two-way repeated measures ANCOVA while adjusting for the following covariates; age, gender, NYHA functional class, and baseline LVEF and 25-OHD.

### *p* < 0.001).

b The model was adjusted for the same covariates except for baseline 25-OHD.

c The model was adjusted for the same covariates except for baseline LVEF.

6-MWD 6-minute walk distance 25-OHD 25-hydroxyvitamin D BNP brain natriuretic peptide DBP diastolic blood pressure LVEF left ventricular ejection fraction SBP systolic blood pressure

A sensitivity analysis by baseline 25-OHD values split at the median (12.4 ng/mL) was applied. It showed no significant difference in plasma renin change between the bottom and upper classes of plasma 25-OHD in VitD group [−34.2 (−109; −11.2) *vs.* −31.7 (−125; −41.7); *p* = 0.526] and PLB group [−1.02 (−3.32; −2.20) *vs.* 3.41 (−1.03; 7.20); *p* = 0.08]. No “time*group” interactions were found for cardiac function and physiological measures (i.e., blood pressure, heart rate, NYHA class, 6-MWD, and LVEF), nor other laboratory parameters including BNP (Table 2). None of the patients demonstrated VitD toxicity signs at the two-week and one-month visits. Plasma calcium and urinary calcium: creatinine ratio did not exceed 2.6 mmol/L and 0.15, respectively.

## Discussion

The study revealed a high prevalence of hypovitaminosis D in these HFrEF patients. It also showed significant “time*group” interactions for 25-OHD and renin plasma concentrations. VitD supplementation resulted in a decrease in renin concentrations in parallel with the increase in 25-OHD levels. Moreover, the changes in renin were inversely related to the changes in 25-OHD. However, no effects were found for clinical, echocardiographic, and other laboratory parameters including BNP.

Previous reports described lower 25-OHD levels in CHF patients compared to healthy subjects^16–18^. The prevalence of hypovitaminosis D of 87.5% in these CHF patients is higher than the general population in Tunisia (47.5%)^19^. However, it is equivalent to prevalence in American CHF patients (89%)^17^. The deficit in VitD might be explained at least partly by poor outdoor activities and low exposure to sunlight in CHF patients.

The RAAS plays a central role in the pathophysiology of CHF^3,20^. Both renin activity and concentration were elevated and associated with a poor prognosis in HFrEF patients^21,22^. In these series, most patients had high plasma renin concentrations.

Evidence from animal and human studies suggests an association of low serum VitD with RAAS upregulation, which signifies that the vitamin exerts inhibitory effects on the system^23,24^. The high rate of hyperreninemia in these CHF patients likely results from the treatment supplied over long periods and from hypovitaminosis D.

The effect of VitD supplementation on the RAAS has been investigated in CHF and other cardiovascular diseases yielding conflicting results. VitD supplementation resulted in a decrease in renin concentration/activity in patients with stable HFrEF^12^, diabetic nephropathy^24^, and stable coronary artery disease^25^. Supplementation for 6 months in CHF patients decreased aldosterone but did not change renin levels^13^. Other clinical trials reported no benefit of supplementation on RAAS in CHF patients^14^ and overweight/obese individuals^26^.

The mechanisms underlying the modulating effect of VitD on the RAAS are not entirely understood. Animal studies showed that VitD deficiency stimulates renin expression whereas injection of calcitriol (1,25(OH)2D3) led to renin suppression^8,27,28^. It has been suggested that increased renin production is due to hyperparathyroidism^29–31^. However, it was demonstrated that renin suppression by calcitriol is independent of parathyroid hormone^8,28,32^.

Experimental evidence suggests that VitD is beneficial in cardiac dysfunction^33–36^. However, RCTs evaluating the effect of VitD supplementation on cardiac function yielded inconsistent results. A meta-analysis concluded that VitD supplementation results in beneficial effects on ventricular hypertrophy and LVEF^7^. However, other meta-analyses concluded that VitD does not affect left ventricular function^37,38^ or improve LVEF, 6-MWD test, and natriuretic peptide levels^39^. Inconsistency is due to factors related to both patients and supplement characteristics.

Patient-related factors include age, gender, comorbidities, LVEF rate, NYHA class, medications, compliance to treatment, degree of RAAS activation, prior vitamin supplement use, and especially baseline VitD status. In this study, no significant differences in plasma renin change were found according to plasma 25-OHD class in both VitD and PLB groups. Most patients have low baseline 25-OHD levels. Elsewhere, VitD supplementation differed by dosing, form, frequency of administration, duration, compliance with supplements, and efficiency in recovering VitD sufficiency. Supplementation would be more effective as it concerns patients with basal deficit and mild/moderate impairment of myocardial function, and as it ensures the restoration of sufficient VitD status.

Previous trials used various regimens of supplements including daily doses of 1000 to 10,000 IU, weekly doses of 50,000 IU, or monthly doses of 400,000 IU (in one trial an initial dose of 300,000 IU then 50,000 IU monthly doses)^7,13,37,38^. Since VitD deficiency is widespread in the Tunisian population^19^, we expected that the deficiency would be highly prevalent and severe in CRF patients, subjects at high risk for deficiency. Hence, we opted for a high dose aiming to restore adequate status and properly evaluate the effect of VitD on RAAS. For efficacy and safety reasons, the supplements were divided into two doses of 200,000 IU supplied at a two-week interval. The regimen ensures reserve stocks filling and a gradual and persistent rise in circulating 25-OHD while avoiding poor compliance and VitD intoxication. Really, in most patients, VitD supplementation resulted in a recovery of sufficient VitD status with no adverse effects.

In the current series, most patients had hypovitaminosis D and highly activated RAAS, and no one had previously taken VitD supplements. Indeed, a long-time VitD intake might confound the effect of time-limited supplementation in RCTs. The study involved clinically stable or stabilized heart failure patients; nearly 90% of patients with NYHA class II and normal BNP concentration. Mild ventricular hypertrophy makes it more probable that VitD has a reparative effect than severe hypertrophy. VitD supplementation failed to induce significant changes in clinical and physiological measures in patients. The null effects might be due to insufficient power to detect significant changes in these outcomes and the short follow-up period. Clinical effects would require longer periods of supplementation and follow-up to become apparent.

Moreover, although supplementation-based studies are viewed as the gold standard of evidence, they have pitfalls. The supplementation is generally limited in time and does not reflect long-standing impregnation of the body. A substance such as VitD is usually supplied in an inactive form that needs ongoing metabolic steps (i.e., intestinal absorption, transport into circulation, sequential hydroxylation, cell uptake, and receptor binding) to gain activity. All these steps might be compromised in CHF patients due to inflammation and heart, liver, and kidney dysfunctions. Finally, VitD is supplied in supra-physiological doses, which could create an imbalance with potential synergistic factors causing metabolic pathways inhibition.

The study has some limitations that require mention. It was a single-center study with most patients having clinically stable or stabilized heart failure. This prevents the generalization of the results to other types of CHF patients. The main limitation of the current trial is the shortness of the follow-up period. The period might not be long enough to cause significant changes in physiological and clinical outcomes. The full effects of repletion on blood pressure, cardiac function, and clinical features may require a longer period of VitD sufficiency. Future research with longer follow-ups is needed to understand the role of VitD on RAAS and cardiac function.

## Conclusions

The study revealed a high prevalence of VitD deficiency in HFrEF patients. A high VitD dose in HFrEF patients resulted in medium-term suppression of SRAA but no effect on cardiac function and clinical outcomes. It should be evaluated whether persistent adequate VitD status in CHF patients results in long-term SRAA suppression and whether this impacts cardiac function and clinical outcomes. VitD is assumed to exert beneficial effects on muscle cells, endothelium, and inflammation, and affects cardiac contraction and remodeling through its action on calcium and parathyroid hormone. Therefore, patients with CHF should be screened for VitD deficiency and have any deficiency corrected. Such a strategy might reduce RAAS activation, prevent/reduce poor outcomes, and improve health. Data from the current trial and literature do not provide sufficient evidence to recommend VitD supplementation in heart failure. Further research is needed to clarify the role of VitD in the field. Specifically, large RCTs with longer follow-ups should be performed to evaluate clinical and echocardiographic impacts of VitD supplementation in heart failure.

## Acknowledgement

The authors thank the patients, the staff of the cardiology department, and the biochemistry laboratory for their contribution.

## Funding

The study was supported by funds from the Committee of Research and Innovation at Rabta Hospital, and Research Laboratory “LR99ES11”, Ministry of Higher Education and Scientific Research of Tunisia.

## Declaration of interest

The authors declare no conflict of interest.
